# Supplementary material for: A machine learning model reveals expansive downregulation of ligand-receptor interactions that enhance lymphocyte infiltration in melanoma with developed resistance to immune checkpoint blockade
Source: Nat Commun. 2024 Oct 14;15:8867. doi: 10.1038/s41467-024-52555-4 (PMC11473774; doi:10.1038/s41467-024-52555-4)
Supplement: Supplementary file 1 — Supplementary Information [file 41467_2024_52555_MOESM1_ESM.pdf]

Title

A machine learning model reveals expansive downregulation of ligand-receptor interactions enhancing lymphocyte infiltration in melanoma with developed resistance to Immune Checkpoint Blockade

Author list

Sahil Sahni[1], Binbin Wang[1], Di Wu[2], Saugato Rahman Dhruba[1], Matthew Nagy[1], Sushant Patkar[3], Ingrid Ferreira[4], Chi-Ping Day[1], Kun Wang[1,5,#], Eytan Ruppin[1,#]

Affiliations

[1]Cancer Data Science Laboratory (CDSL), Center for Cancer Research (CCR), National Cancer Institute (NCI), National Institutes of Health (NIH), Bethesda, MD USA

[2]Laboratory of Pathology, Center for Cancer Research (CCR), National Cancer Institute (NCI), National Institutes of Health (NIH), Bethesda, MD USA

[3]Artificial Intelligence Resource, Molecular Imaging Branch, National Cancer Institute (NCI), National Institutes of Health (NIH), Bethesda, MD USA

[4]Experimental Cancer Genetics, Wellcome Sanger Institute, Wellcome Genome Campus, Hinxton, Cambridge UK

[5]Department of Comparative Biosciences, University of Illinois Urbana-Champaign, Urbana, IL USA

[#]These authors jointly supervised this work

Corresponding authors: Kun Wang (kwang222@illinois.edu) and Eytan Ruppin (eytan.ruppin@nih.gov)

Supplementary Figures

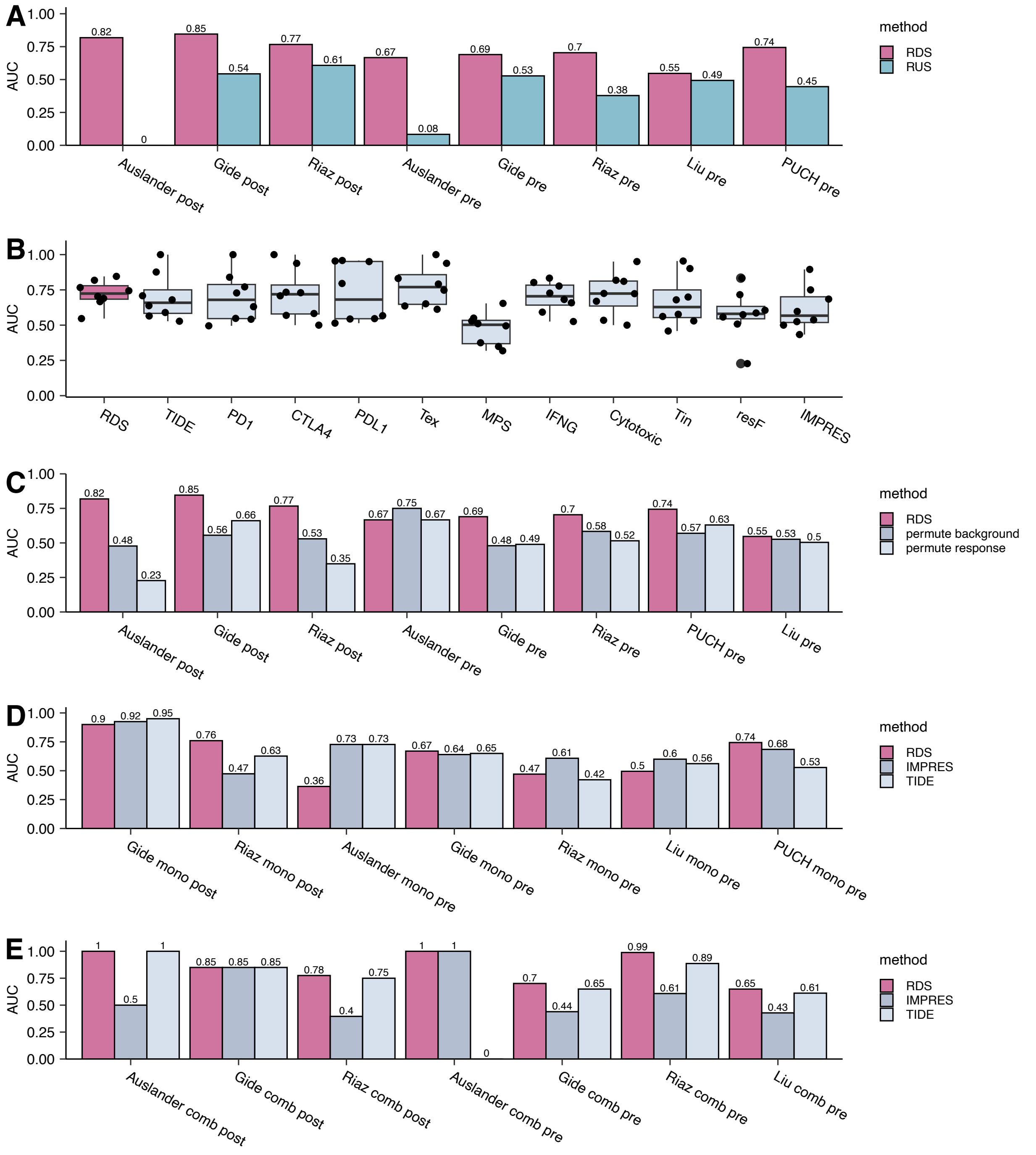

Supplementary Figure 1: Benchmarking RDS-based prediction of ICB response in melanoma. (A) Bar plot depicting AUC in classifying responder vs. non-responder melanoma samples (n=400) between resistance downregulated (RDS) and resistance upregulated (RUS) scores. (B) Boxplot depicting the distribution of AUC in classifying responder vs. non-responder samples in all melanoma ICB cohorts (n=8) between RDS and 11 relevant transcriptomic signatures of ICB and immune response: TIDE (1), *PD1*, *CTLA4*, *PDL1*, T cell exhaustion (Tex), Melanocytic plasticity score (MPS) (2), IFNG signature (3), Cytotoxic signatures (4), T cell inflamed GEP (Tin) (5),

resF (6), IMPRES (7). (C) Bar plot depicting AUC in classifying responder vs. non-responder melanoma samples (n=400) between RDS, RDS when permuting cell-type-specific ligand-receptor interaction profile, and RDS when permuting patient response. (D) Bar plot depicting AUC in classifying responder vs. non-responder melanoma ICB monotherapy (anti-PD1 or anti-CTLA4) samples (n=233) between RDS, TIDE (1), and IMPRES (7). (E) Bar plot depicting AUC in classifying responder vs. non-responder melanoma ICB combination therapy (anti-PD1 with anti-CTLA4) samples (n=146) between RDS, TIDE (1), and IMPRES (7).

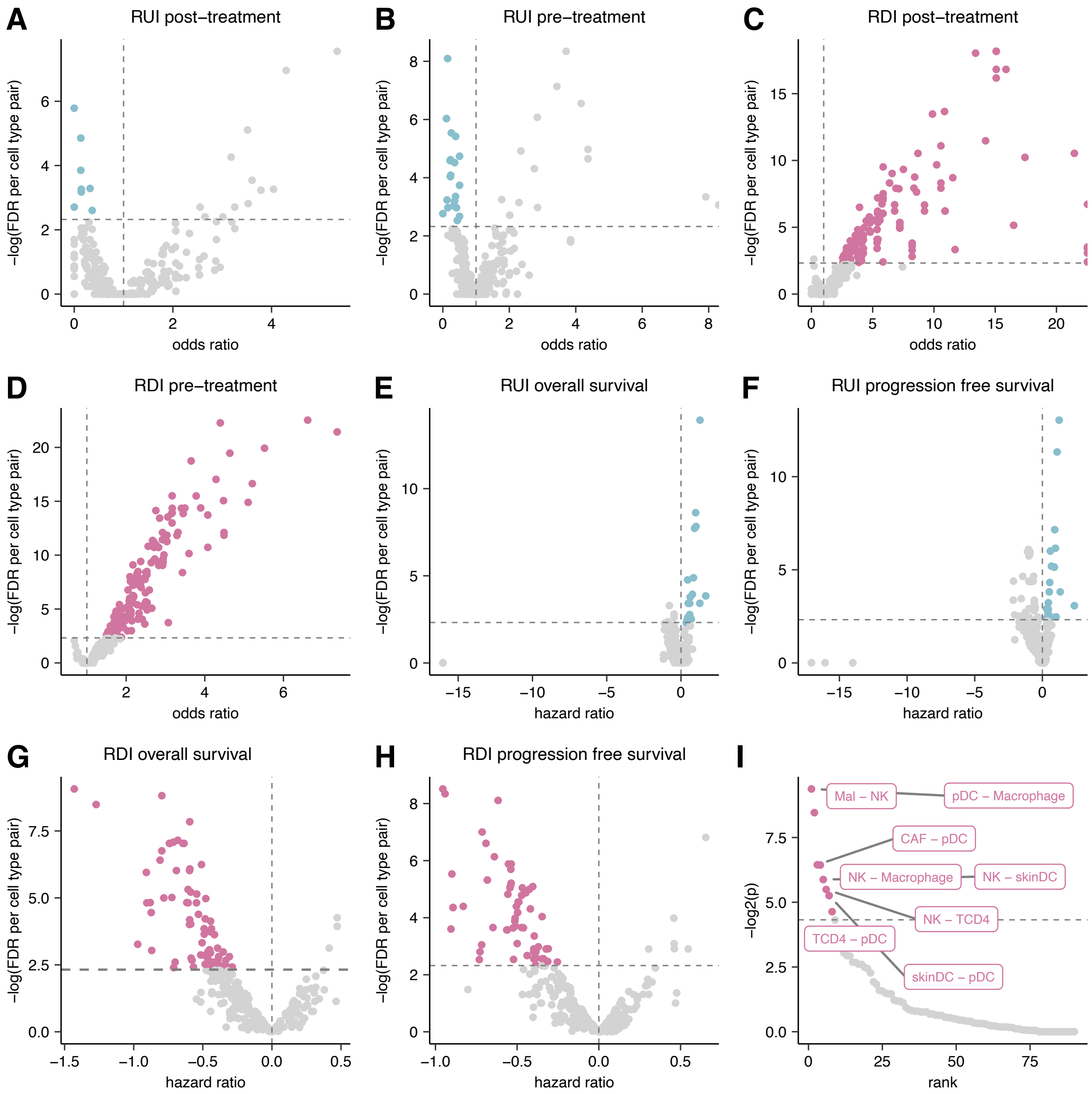

Supplementary Figure 2: The association between individual RDI interactions and patient response to ICB and survival. (A-D) Enrichment analysis depicting individual activated RUIs (A-B) and RDIs (C-D) in responder versus

non-responder patients among post-treatment (A and C) or pre-treatment (B and D) samples (n_{post}=90, n_{pre}=310). The X-axis indicates the odds-ratio of enrichment (responder > 1, non-responder < 1), and the Y-axis indicates the significance (FDR per cell type pair) from Fisher's two-sided test. (A-B) RUIs with an odds-ratio < 1 and FDR < 0.2 per cell type pair are considered significantly activated in non-responder samples and are highlighted in blue. (C-D) RDIs with an odds-ratio > 1 and FDR < 0.2 per cell type pair are considered significantly activated in responder and are highlighted in magenta. (E-H) Univariable cox proportional hazard regression analysis identified individual activated RUIs (E-F) or RDIs (G-H) in the combined set of pre-treatment samples receiving ICB therapy with overall survival (E and G) and progression free (F and H) survival timelines (n_{OS}=296, n_{PFS}=241). The X-axis indicates the hazard ratio of individual interactions based on their activity (activated or inactivated) either providing beneficial survival outcome (< 0) or adverse survival outcome (> 0). The Y-axis indicates the significance (FDR per cell type pair) of individual interactions hazard ratios. (E-F) RUIs with a hazard ratio > 0 and FDR < 0.2 per cell type pair are considered significantly associated with adverse overall and progression free survival outcome respectively in pre-treatment samples and are highlighted in blue. (G-H) RDIs with a hazard ratio < 0 and FDR < 0.2 per cell type pair are considered significantly associated with beneficial overall and progression free survival outcome respectively in pre-treatment samples and are highlighted in magenta. (I) Enrichment analysis depicting top ranked ligand cell-receptor cell pairs enriched within the RDI network. Background were all LIRICS' tumor-immune interactions inferred (n=3776). The Y-axis indicates the *p*-value from the cell pair enrichment analysis. The X-axis indicates the rank in ascending order starting from the most significantly enriched cell-pair. Cell pairs with *p*-value < 0.05 (dotted line) were considered significantly enriched and are highlighted in magenta.

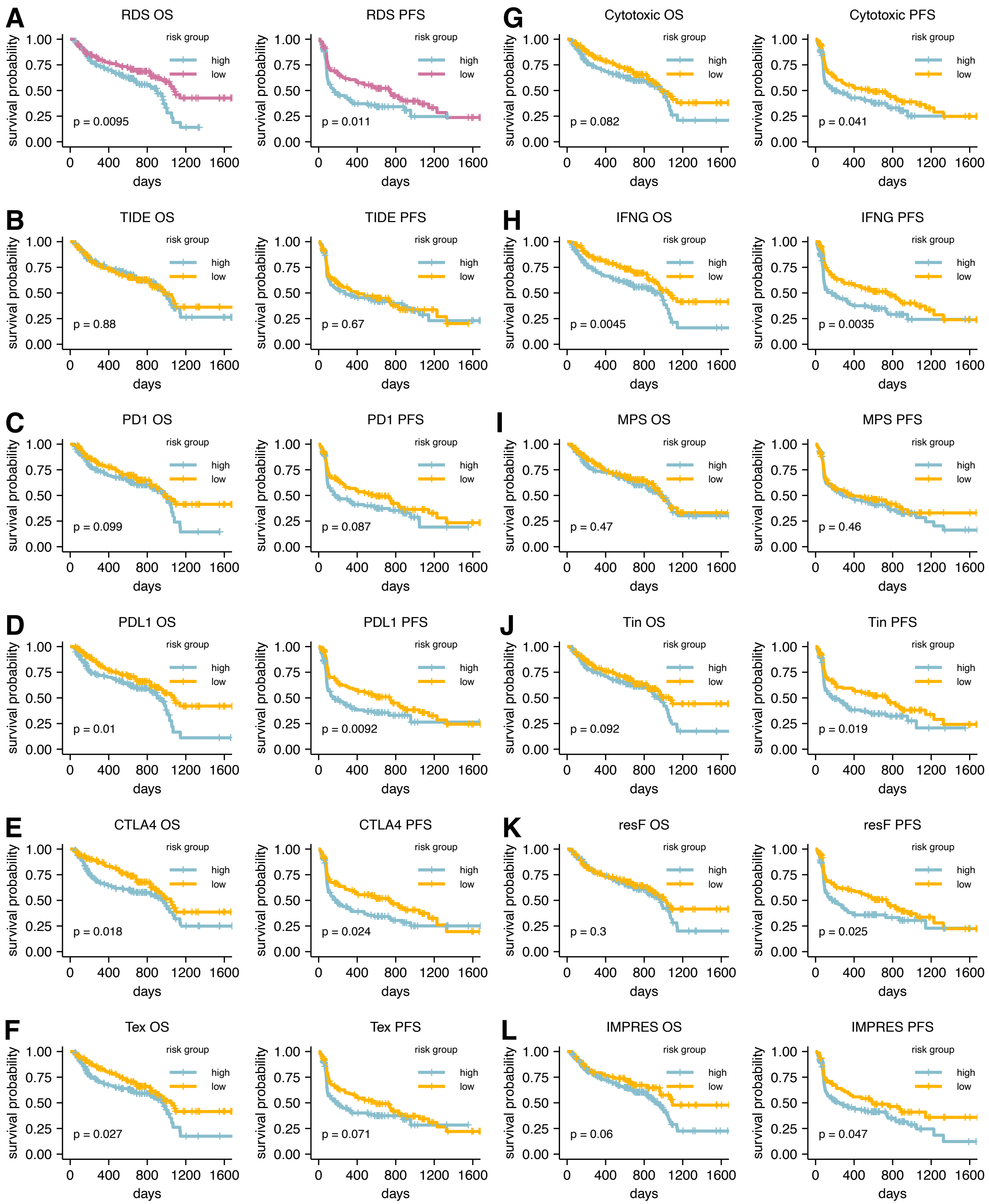

Supplementary Figure 3: Comparative Survival stratification performance of RDS versus eleven transcriptomics-based biomarkers in melanoma patients receiving ICB. Survival stratification performance of RDS score vs. other relevant bulk-transcriptomics signatures of the combined set of pre-treatment melanoma samples (n_{OS}=296, n_{PFS}=241) receiving immune checkpoint blockade. (A) Kaplan-Meier plots depicting progression free (PFS)

and overall survival (OS) differences between the low and high-risk groups defined by the median value of RDS. The significance of survival differences was estimated using the log-rank test. Time on the X-axis is measured in days. (B-L) Kaplan-Meir plots showing PFS and OS differences between low and high-risk groups defined by the median value of relevant transcriptomic signatures. The significance of survival differences was estimated using the log-rank test. Time on the X-axis is measured in days. The signatures evaluated in each panel are: (B) TIDE (1), (C) *PD1*, (D) *CTLA4*, (E) *PDL1*, (F) T cell exhaustion (Tex), (G) Melanocytic plasticity score (MPS) (2), (H) IFNG signature (3), (I) Cytotoxic signatures (4), (J) T cell inflamed GEP (Tin) (5), (K) resF (6), (L) IMPRES (7).

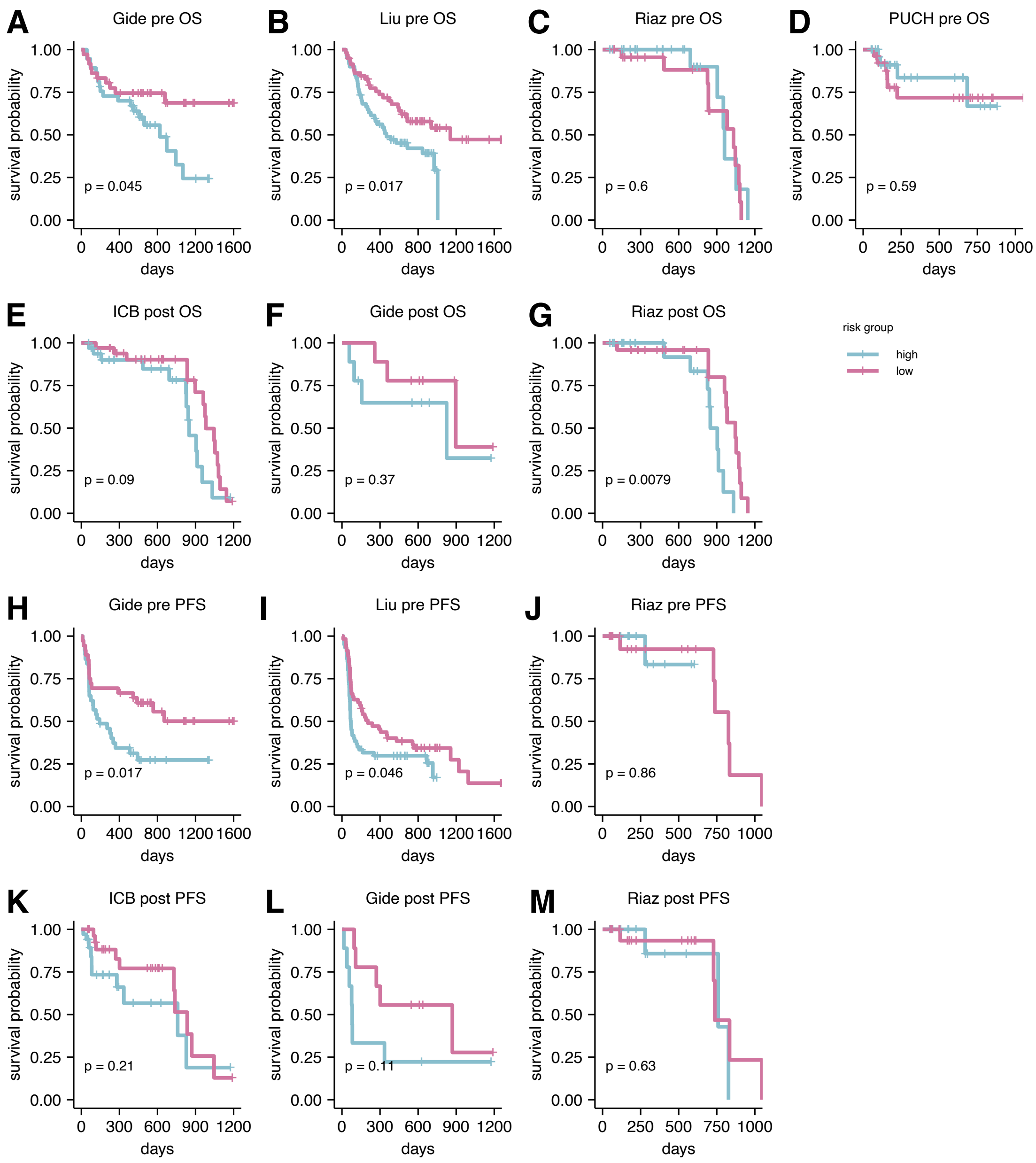

Supplementary Figure 4: Survival stratification performance of RDS in individual melanoma ICB cohorts. Survival stratification performance of RDS for melanoma patients receiving checkpoint therapy. Kaplan-Meir plots showing progression free survival (PFS) and overall survival (OS) differences between low (in magenta) and high-risk (in blue) groups defined by the median value of RDS. The significance of survival differences was estimated using the log-rank test. Time on the X-axis is measured in days. (A-D) Kaplan-Meir plots depicting OS of individual checkpoint cohorts' pre-treatment patients. n (from left to right): 73, 119, 49, and 55. (E) Kaplan-Meir plots depicting OS of the combined set post-treatment checkpoint treated patients (n=67). (F-G) Kaplan-Meir plots depicting OS of individual checkpoint cohorts' post-treatment patients. n (from left to right): 18 and 49. (H-J) Kaplan-Meir plots depicting PFS of individual checkpoint cohorts' pre-treatment patients. n (from left to right): 73, 119, and 49. (K) Kaplan-Meir plots depicting PFS of the combined set post-treatment checkpoint treated patients (n=67). (L-M)

Kaplan-Meir plots depicting PFS of individual checkpoint cohorts' post-treatment patients. n (from left to right): 18 and 49.

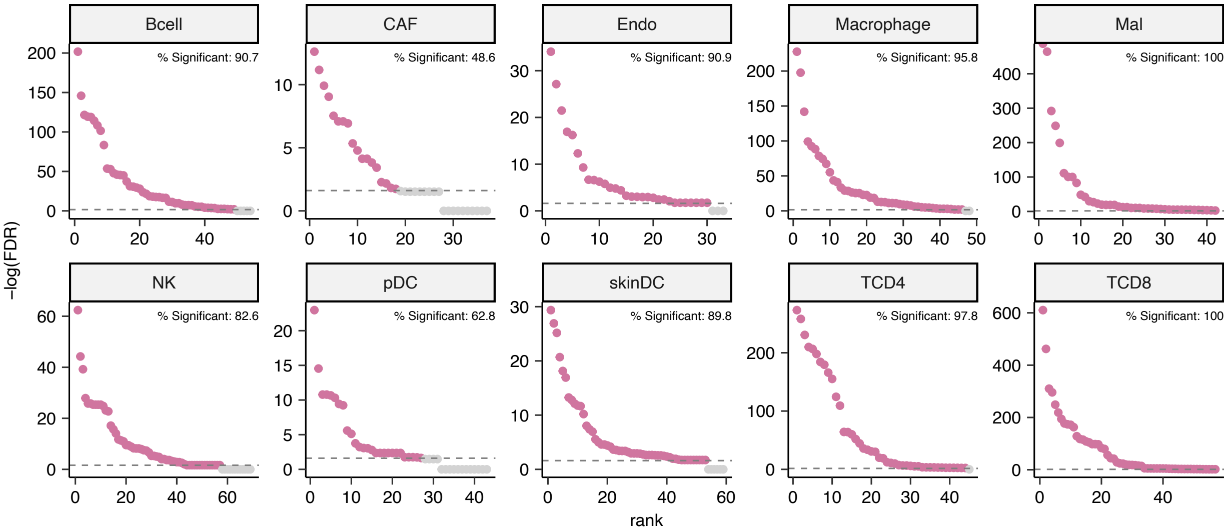

Supplementary Figure 5: Validation of ligand and receptor expression in relevant cell types for RDIs. Dot plots depicting the expression landscape of ligand and receptor genes within each respective cell type. The Y-axis denotes the negative log of the false discovery rate (FDR) obtained from the one-sample t-test for each ligand and receptor gene expression within each cell type. The X-axis lists the significantly expressed ligand or receptor gene within the respective cell type in ascending order. Ligand and receptor genes with an FDR < 0.2 (indicated by the dotted line) were deemed significantly expressed and are highlighted in magenta. The percentage of significant ligand and receptor genes relative to all inferred genes within the RDI network is provided in the top right corner of each plot. The respective cell type is highlighted at the top of each plot.

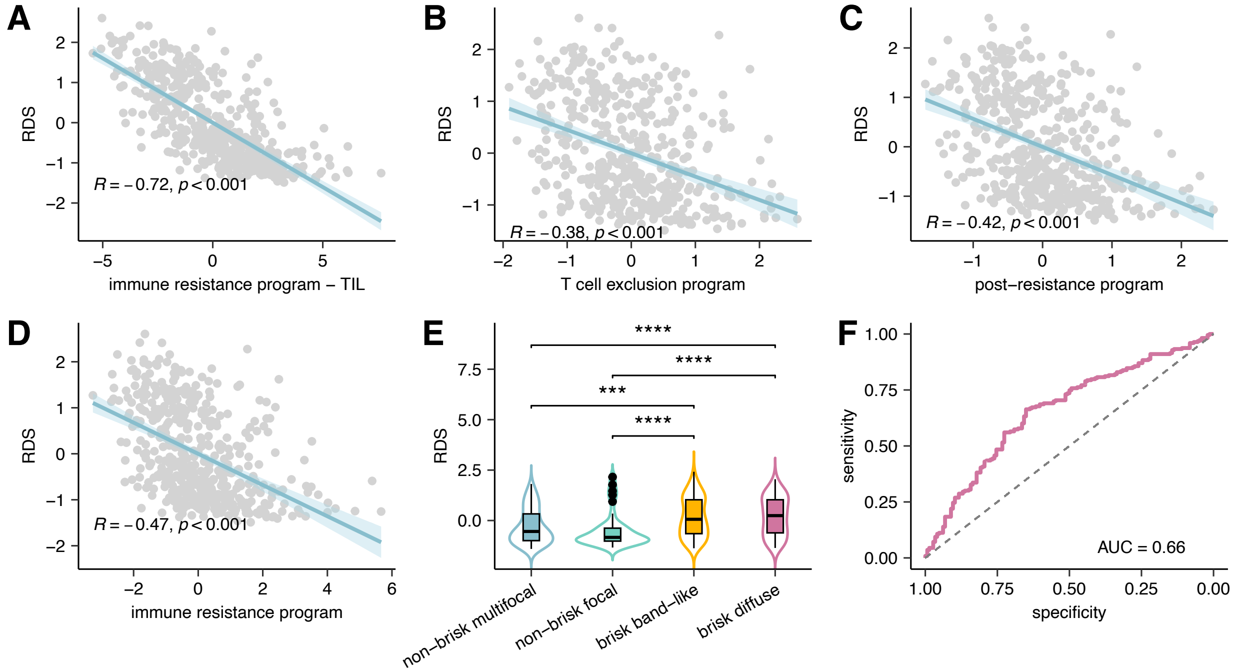

Supplementary Figure 6: The association between RDS scores and ICB resistance gene programs and T cells infiltration in the TME. (A) Scatter plot depicting the correlation the between RDS and transcriptomic signatures of immune resistance in TCGA-SKCM. The immune resistance program includes the combined set of genes inferred for both the T cell exclusion and post-resistance programs. The X-axis indicates the immune resistance program adjusted for TIL. Pearson R = 0.72, $P = 3.3 \times 10^{-75}$ (n=468). (B) Scatter plot depicting the correlation the between RDS and transcriptomic signatures of T cell exclusion program in TCGA-SKCM. Pearson R = -0.38, $P = 2.5 \times 10^{-17}$ (n=468). (C) Scatter plot depicting the correlation the between RDS and transcriptomic signatures of post-resistance program in TCGA-SKCM. Pearson R = -0.42, $P = 2.9 \times 10^{-21}$ (n=468). (D) Scatter plot depicting the correlation the between RDS and transcriptomic signatures of immune resistance program in TCGA-SKCM. The immune resistance program includes the combined set of genes inferred for both the T cell exclusion and post-resistance programs. Pearson R = -0.47, $P = 9.5 \times 10^{-27}$ (n=468). (E) Boxplot depicting distribution of RDS between non-brisk and brisk subtypes in TCGA-SKCM (n=369). One-sided Wilcoxon test *p*-values (from top to bottom): 8.9×10^{-5}, 7.9×10^{-5}, 0.00011, and 5.9×10^{-5}. The boxplot displays median, 25 and 75 percentiles (Q1 and Q3) as bounds of the box, and whiskers that extends from the box to a minima of Q1 - 1.5 × IQR and maxima Q3 + 1.5 × IQR (where IQR is interquartile range). (F) ROC curve depicting classifying hot vs. cold tumor niches in TCGA-SKCM using RDS.

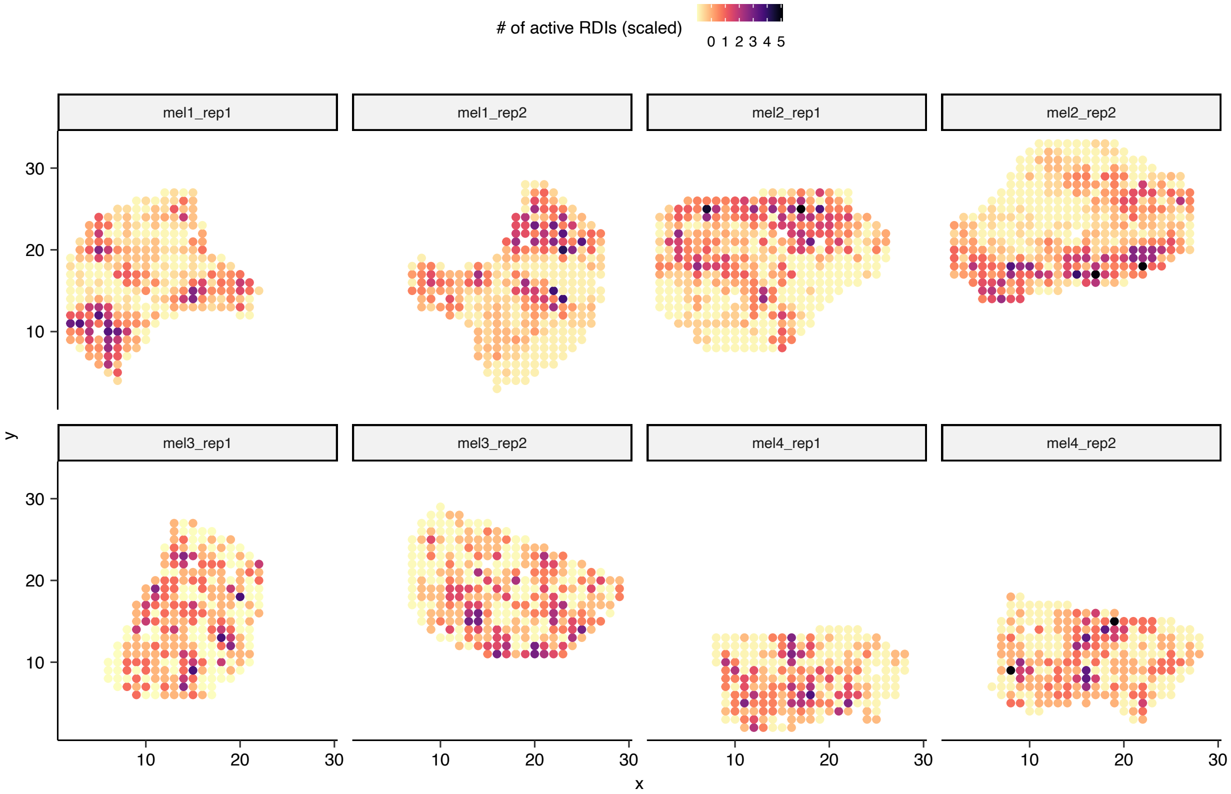

Supplementary Figure 7: Spatial distribution of RDIs in treatment naïve metastatic melanoma biopsies. Scatter plot illustrating the number of activated RDIs (scaled) within each spatial region of legacy spatial transcriptomics slides (n=8). The X-axis corresponds to the x-coordinate of the spatial region, and the Y-axis corresponds to the y-coordinate of the spatial region.

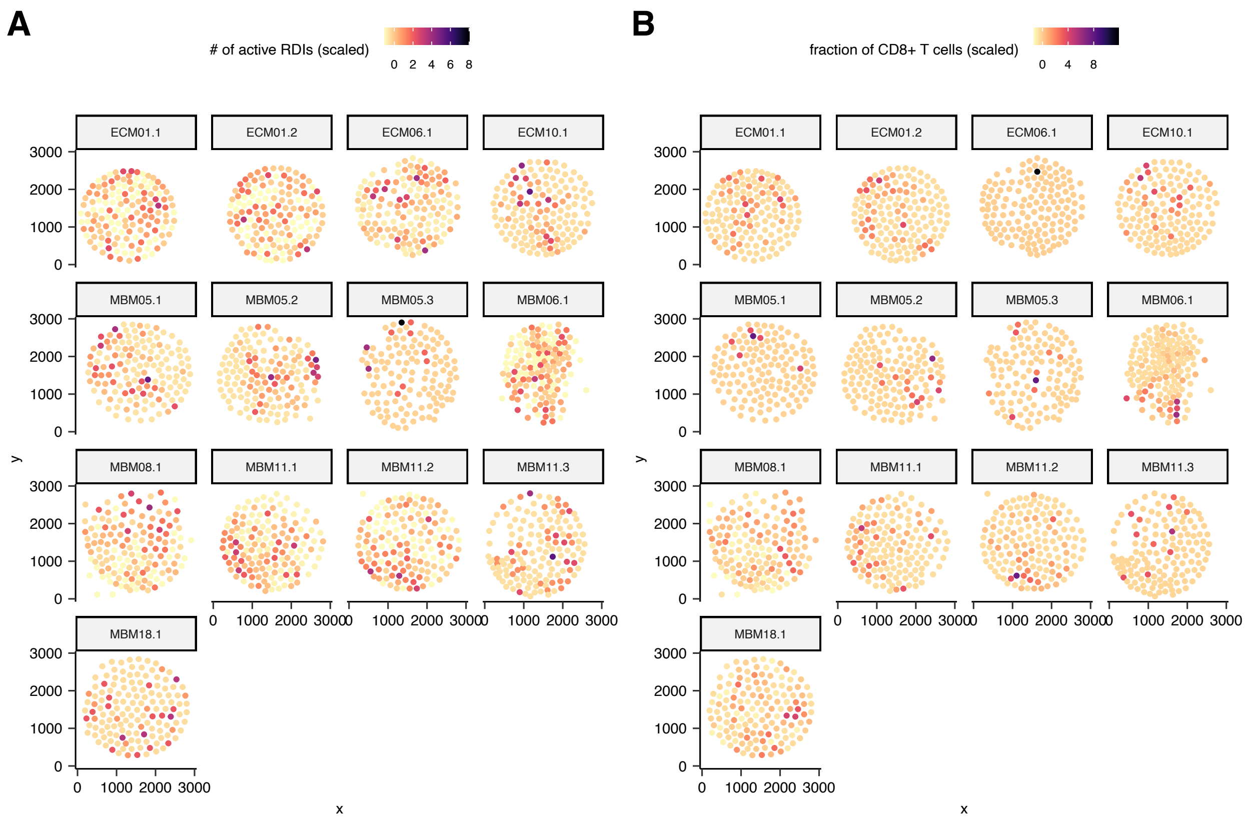

Supplementary Figure 8: The spatial association between RDIs and CD8+ T cells in metastatic melanoma biopsies. (A) Scatter plot illustrating the number of activated RDIs (scaled) within each spatial region of SlideSeqV2 spatial transcriptomics slides (n=13). The X-axis corresponds to the x-coordinate of the spatial region, and the Y-axis corresponds to the y-coordinate of the spatial region. (B) Corresponding scatter plots depicting the fraction of CD8+ T cell infiltration (scaled) within each spatial region of the SlideSeqV2 slides (n=13).

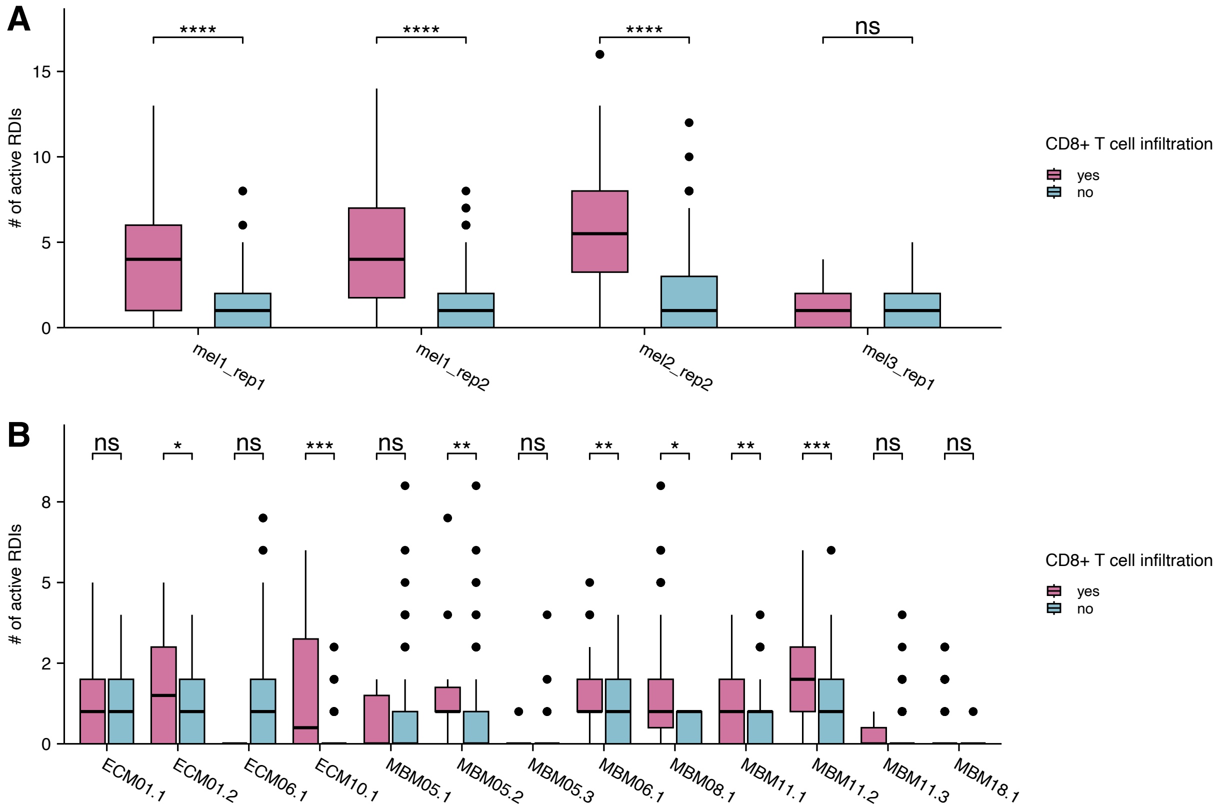

Supplementary Figure 9: RDIs are associated with CD8+ T cell infiltration across different regions in individual spatial transcriptomics slides. (A) Boxplot depicting the total number of activated RDIs between spatial regions with and without CD8+ T cell infiltration at a slide level, based on legacy spatial transcriptomics slides (n=4). One-sided Wilcoxon test *p*-values (from left to right): 1.29×10^{-15} ($n_{regions}$=277), 3.55×10^{-16} ($n_{regions}$=292), 9.51×10^{-20} ($n_{regions}$=380), and 0.73 ($n_{regions}$=255). (B) Boxplot depicting the total number of activated RDIs between spatial regions with and without CD8+ T cell infiltration at a slide level, based on SlideSeqV2 spatial transcriptomics slides (n=13). One-sided Wilcoxon test *p*-values (from left to right): 0.46 ($n_{regions}$=144), 0.013 ($n_{regions}$=144), 0.86 ($n_{regions}$=144), 0.00048 ($n_{regions}$=143), 0.53 ($n_{regions}$=140), 0.0010 ($n_{regions}$=142), 0.059 ($n_{regions}$=126), 0.0037 ($n_{regions}$=144), 0.011 ($n_{regions}$=144), 0.0065 ($n_{regions}$=144), 0.00030 ($n_{regions}$=144), 0.048 ($n_{regions}$=144), and 0.38 ($n_{regions}$=143). The boxplots for figures (A) and (B) displays median, 25 and 75 percentiles (Q1 and Q3) as bounds of the box, and whiskers that extends from the box to a minima of Q1 - 1.5 × IQR and maxima Q3 + 1.5 × IQR (where IQR is interquartile range).

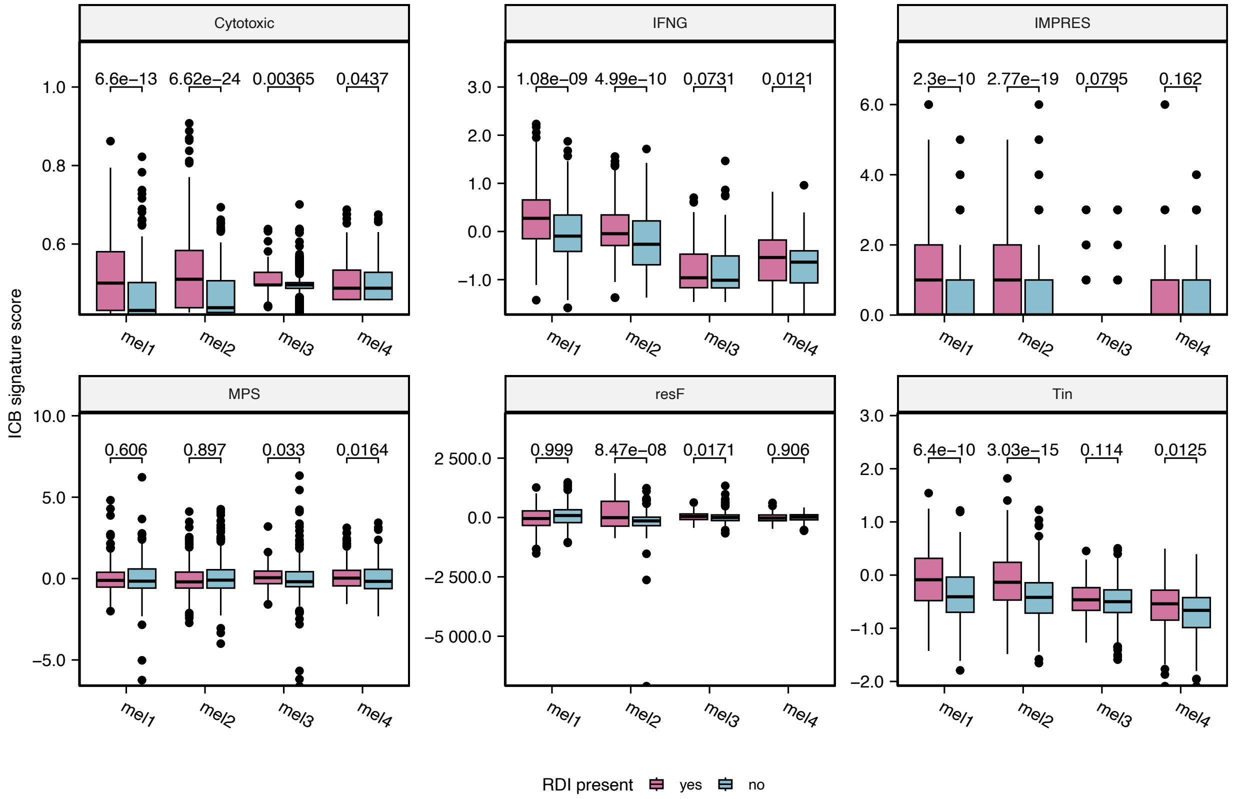

Supplementary Figure 10: RDI activity is associated with ICB response biomarkers in spatial transcriptomics. Box plot illustrating the distribution of six ICB signature scores between spatial regions with and without any RDIs present at a patient level (n=4), based on legacy spatial transcriptomics slides (n=8). The signatures evaluated in each panel are: Cytotoxic signature (4), IFNG signature (3), IMPRES (7), MPS (2), resF (6), and T cell inflamed GEP (Tin) (5). The original MPS score was adjusted by multiplying the score by negative one so that greater adjusted MPS scores are associated with ICB responders for the purpose of visualization. Significance derived from one-sided Wilcoxon test. The boxplot displays median, 25 and 75 percentiles (Q1 and Q3) as bounds of the box, and whiskers that extends from the box to a minima of Q1 - 1.5 × IQR and maxima Q3 + 1.5 × IQR (where IQR is interquartile range).

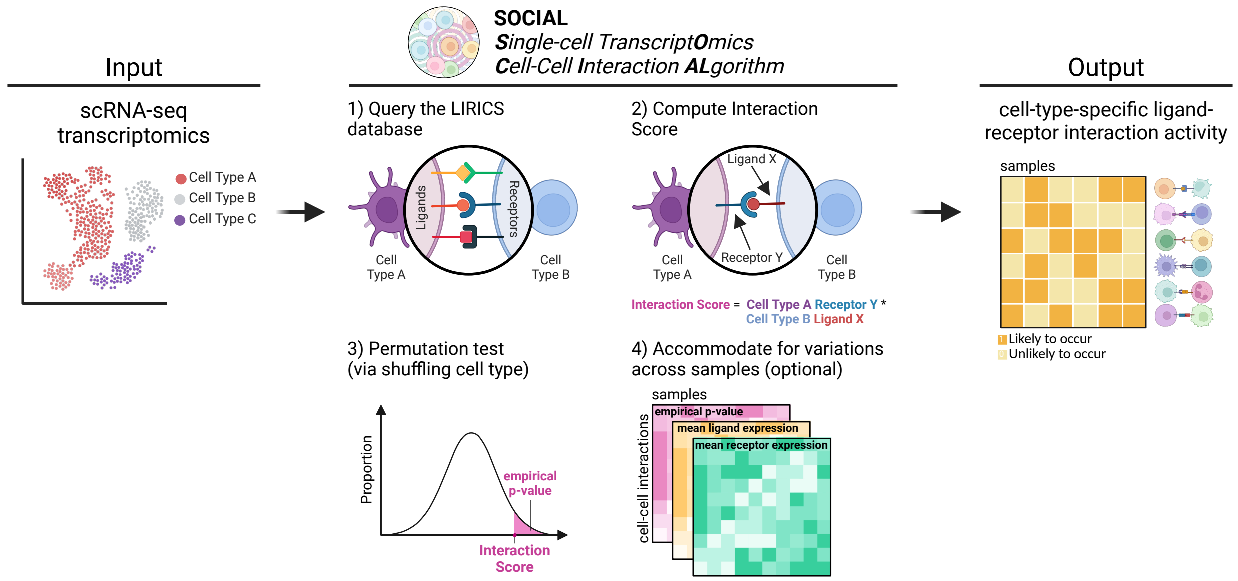

Supplementary Figure 11: Overview of SOCIAL. The SOCIAL input includes both the single-cell transcriptomics expression data and annotated cell type information. It consists of three major steps: Step I query the LIRICS database to identify plausible ligand-receptor interactions. Step II computes an interaction score by multiplying the average expression levels of the ligand and receptor complexes for each interaction pair and cell type. Step III performs a permutation test by shuffling cell type labels to derive an empirical *p*-value. Optionally in Step IV, ligand-receptor interactions can be further denoted as significantly activated if the average expression level of both the ligand and receptor genes within the respective cell type is greater than the median across all samples. The final output of SOCIAL is a cell-type-specific ligand-receptor interaction activity profile across all samples. Supplementary Figure 11, created with BioRender.com, released under a Creative Commons Attribution-NonCommercial-NoDerivs 4.0 International license https://creativecommons.org/licenses/by-nc-nd/4.0/deed.en.

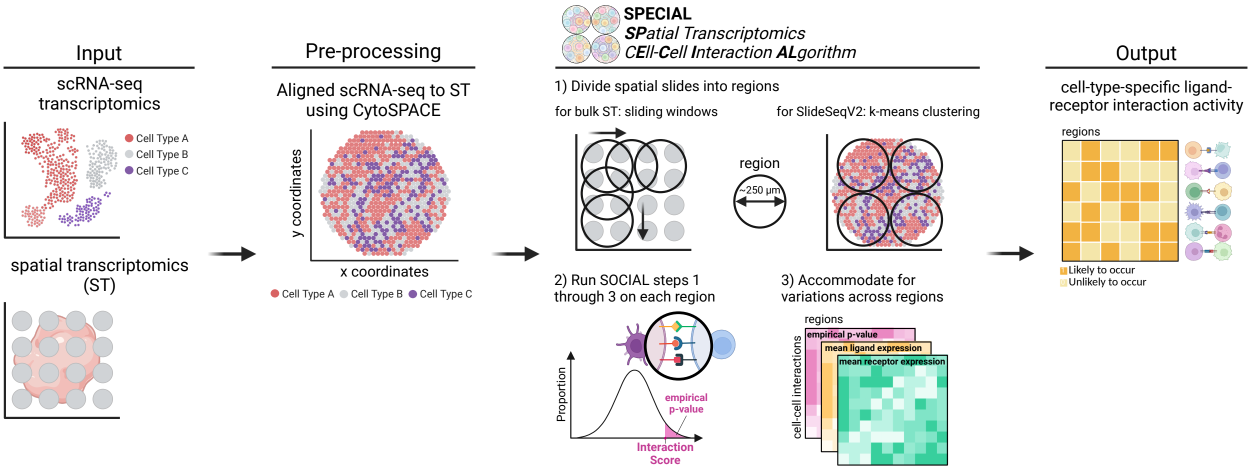

Supplementary Figure 12: Overview of SPECIAL. The SPECIAL input is the aligned single-cell transcriptomics to spatial transcriptomics (inferred by applying CytoSPACE to single-cell and spatial transcriptomics). It consists of three major steps: Step I utilizes either a sliding window or k-means clustering approach on bulk (i.e. Visium 10X and Legacy) and SlideSeqV2 spatial transcriptomics, respectively, to divide spatial slides into "regions" of approximately 250 µm in diameter. Step II employs SOCIAL steps 1 through 3 to infer cell-type-specific interaction activity within each ~250 µm region. Step III, ligand-receptor interactions are further denoted as significantly activated if the average expression levels of both the ligand and receptor genes within the respective cell type is greater than the median across all regions. The final output of SPECIAL is a cell-type-specific ligand-receptor interaction activity profile across all regions in a spatial transcriptomics slide. Supplementary Figure 12, created with BioRender.com, released under a Creative Commons Attribution-NonCommercial-NoDerivs 4.0 International license https://creativecommons.org/licenses/by-nc-nd/4.0/deed.en.

Original Cell Type	Reannotated Cell Type
Plasma cells	Bcell
Tumor cells	Mal
Dendritic cells	skinDC
Microglia	Macrophage
MDM	Macrophage
Monocytes	Additional-Immune
Tregs	Additional-Immune
CD8+ T cells	TCD8
B cells	Bcell
NK cells	NK
CD4+ T cells	TCD4
CNS cells	CNS
Stromal cells	CAF
Endothelial cells	Endo
Mast cells	Additional-Immune
Epithelial cells	Epithelial

Supplementary Table 1: Manual reannotation of cell types for Biermann et al.'s snRNA-seq cohort.

Reference

1. Jiang P, Gu S, Pan D, Fu J, Sahu A, Hu X, et al. Signatures of T cell dysfunction and exclusion predict cancer immunotherapy response. Nat Med [Internet]. 2018;24(10):1550–8. Available from: https://doi.org/10.1038/s41591-018-0136-1

2. Pérez-Guijarro E, Yang HH, Araya RE, El Meskini R, Michael HT, Vodnala SK, et al. Multimodel preclinical platform predicts clinical response of melanoma to immunotherapy. Nat Med [Internet]. 2020;26(5):781–91. Available from: https://doi.org/10.1038/s41591-020-0818-3

3. Ayers M, Lunceford J, Nebozhyn M, Murphy E, Loboda A, Kaufman DR, et al. IFN-γ–related mRNA profile predicts clinical response to PD-1 blockade. J Clin Invest [Internet]. 2017 Aug 1;127(8):2930–40. Available from: https://doi.org/10.1172/JCI91190

4. Davoli T, Uno H, Wooten EC, Elledge SJ. Tumor aneuploidy correlates with markers of immune evasion and with reduced response to immunotherapy. Science (1979) [Internet]. 2017 Jan 20;355(6322):eaaf8399. Available from: https://doi.org/10.1126/science.aaf8399

5. Steiniche T, Rha SY, Chung HC, Georgsen JB, Ladekarl M, Nordsmark M, et al. Prognostic significance of T-cell–inflamed gene expression profile and PD-L1 expression

in patients with esophageal cancer. Cancer Med [Internet]. 2021 Dec 1;10(23):8365–76. Available from: https://doi.org/10.1002/cam4.4333

6. Jerby-Arnon L, Shah P, Cuoco MS, Rodman C, Su MJ, Melms JC, et al. A Cancer Cell Program Promotes T Cell Exclusion and Resistance to Checkpoint Blockade. Cell [Internet]. 2018;175(4):984-997.e24. Available from: https://www.sciencedirect.com/science/article/pii/S0092867418311784

7. Auslander N, Zhang G, Lee JS, Frederick DT, Miao B, Moll T, et al. Robust prediction of response to immune checkpoint blockade therapy in metastatic melanoma. Nat Med [Internet]. 2018;24(10):1545–9. Available from: https://doi.org/10.1038/s41591-018-0157-9
