# Supplementary material for: A machine learning model reveals expansive downregulation of ligand-receptor interactions that enhance lymphocyte infiltration in melanoma with developed resistance to immune checkpoint blockade
Source: Nat Commun. 2024 Oct 14;15:8867. doi: 10.1038/s41467-024-52555-4 (PMC11473774; doi:10.1038/s41467-024-52555-4)
Supplement: Supplementary file 3 — Reporting Summary [file 41467_2024_52555_MOESM3_ESM.pdf]

nature portfolio

Corresponding author(s): Kun Wang and Eytan Ruppin

Last updated by author(s): Aug 7, 2024

Reporting Summary

Nature Portfolio wishes to improve the reproducibility of the work that we publish. This form provides structure for consistency and transparency in reporting. For further information on Nature Portfolio policies, see our Editorial Policies and the Editorial Policy Checklist.

Statistics

For all statistical analyses, confirm that the following items are present in the figure legend, table legend, main text, or Methods section.

n/a	Confirmed	
☐	☒	The exact sample size (*n*) for each experimental group/condition, given as a discrete number and unit of measurement
☐	☒	A statement on whether measurements were taken from distinct samples or whether the same sample was measured repeatedly
☐	☒	The statistical test(s) used AND whether they are one- or two-sided<br>*Only common tests should be described solely by name; describe more complex techniques in the Methods section.*
☒	☐	A description of all covariates tested
☐	☒	A description of any assumptions or corrections, such as tests of normality and adjustment for multiple comparisons
☐	☒	A full description of the statistical parameters including central tendency (e.g. means) or other basic estimates (e.g. regression coefficient) AND variation (e.g. standard deviation) or associated estimates of uncertainty (e.g. confidence intervals)
☐	☒	For null hypothesis testing, the test statistic (e.g. *F*, *t*, *r*) with confidence intervals, effect sizes, degrees of freedom and *P* value noted<br>*Give P values as exact values whenever suitable.*
☒	☐	For Bayesian analysis, information on the choice of priors and Markov chain Monte Carlo settings
☒	☐	For hierarchical and complex designs, identification of the appropriate level for tests and full reporting of outcomes
☐	☒	Estimates of effect sizes (e.g. Cohen's *d*, Pearson's *r*), indicating how they were calculated

Our web collection on statistics for biologists contains articles on many of the points above.

Software and code

Policy information about availability of computer code

Data collection	R (v4.4.1) was used for data collection and organization
Data analysis	IRIS, SOCIAL, and SPECIAL were developed on R (v4.4.1) using R packages: dplyr (v1.1.4), magrittr (v2.0.3), parallel (v4.4.1), pROC (v1.18.5), rBayesianOptimization (v1.2.1), tidyr (v1.3.1), abind (v1.4-5), Matrix (v1.7-0), purrr (v1.0.2), reshape2 (1.4.4), rslurm (v0.6.2), and stats (v4.4.1). All analyses were done on R (v4.4.1).

For manuscripts utilizing custom algorithms or software that are central to the research but not yet described in published literature, software must be made available to editors and reviewers. We strongly encourage code deposition in a community repository (e.g. GitHub). See the Nature Portfolio guidelines for submitting code & software for further information.

Data

Policy information about availability of data

All manuscripts must include a data availability statement. This statement should provide the following information, where applicable:

- Accession codes, unique identifiers, or web links for publicly available datasets
- A description of any restrictions on data availability
- For clinical datasets or third party data, please ensure that the statement adheres to our policy

Data Availability:
The bulk RNA-seq data, CODEFACS' deconvolved expression and cell fraction data for Gide et al. (16), Riaz et al. (18), Liu et al. (17), and TCGA-SKCM from Wang et al.

(15) are available via Zenodo repository (https://zenodo.org/records/5790343). TCGA-SKCM's survival timelines are available from the UCSC Xena browser (https://xenabrowser.net), and pathology classifications are available from Saltz et al. (33). The bulk RNA-seq data for Auslander et al. (9) are available from GEO under the accession number GSE115821 [https://www.ncbi.nlm.nih.gov/geo/query/acc.cgi?acc=GSE115821], and for PUCH (19) from GitHub (https://github.com/xmuyulab/ims_gene_signature). Single-cell RNA-seq data from Jerby-Arnon et al. (12) are available from GEO under the accession number GSE115978 [https://www.ncbi.nlm.nih.gov/geo/query/acc.cgi?acc=GSE115978], with additional cell meta information sourced from TISCH2 (http://tisch.comp-genomics.org). The spatial RNA-seq data from Thrane et al. (37) are available from (https://www.spatialresearch.org/resources-published-datasets/). For Biermann et al. (36), both single-nuclei and spatial RNA-seq data are available from GEO under the accession number GSE185386 [https://www.ncbi.nlm.nih.gov/geo/query/acc.cgi?acc=GSE185386]. The CODEFACS, LIRICS, SOCIAL, and SPECIAL data (and relevant inputs) generated in this study have been deposited in Zenodo repository (https://zenodo.org/records/13172848). Source data are provided with this paper. The remaining data are available within the Article, Supplementary Information, or Source Data file.

Code Availability:
The tools (IRIS, SOCIAL, and SPECIAL) and codes for reproducing the results of this study are available via GitHub (https://github.com/kwangcb/IRIS). Deconvolution tools CODEFACS and LIRICS are available via Zenodo repository (https://zenodo.org/record/5790343). CytoSPACE (v1.0.6) is available via GitHub (https://github.com/digitalcytometry/cytospace).

Research involving human participants, their data, or biological material

Policy information about studies with human participants or human data. See also policy information about sex, gender (identity/presentation), and sexual orientation and race, ethnicity and racism.

Reporting on sex and gender	not applicable.
Reporting on race, ethnicity, or other socially relevant groupings	not applicable.
Population characteristics	not applicable.
Recruitment	not applicable.
Ethics oversight	not applicable.

Note that full information on the approval of the study protocol must also be provided in the manuscript.

Field-specific reporting

Please select the one below that is the best fit for your research. If you are not sure, read the appropriate sections before making your selection.

[x] Life sciences [ ] Behavioural & social sciences [ ] Ecological, evolutionary & environmental sciences

For a reference copy of the document with all sections, see nature.com/documents/nr-reporting-summary-flat.pdf

Life sciences study design

All studies must disclose on these points even when the disclosure is negative.

Sample size	All of the samples were downloaded from public databases. We used the five largest bulk RNA-seq Melanoma treated with immune checkpoint blockade available (Gide et al., Riaz et al., Liu et al., Auslander et al., PUCH) totaling ~400 patients to independently test our approach. We further validated our approach and findings using new untrained deconvolved bulk, single-cell, and spatial transcriptomics datasets (TCGA-SKCM, Jerby-Arnon et al., Thrane et al., Biermann et al.).
Data exclusions	No datasets were excluded.
Replication	We independently validated our approach in five different deconvolved bulk RNA-seq cohorts of immune checkpoint blockade treated melanoma patients. In addition we further validated our findings in one deconvolved bulk RNA-seq melanoma cohort, one single-cell RNA-seq melanoma cohort treated with immune checkpoint blockade, and two spatial transcriptomics data of metastatic melanoma biopsies.
Randomization	Randomization was not relevant to this study.
Blinding	Blinding was not relevant to this study.

Reporting for specific materials, systems and methods

We require information from authors about some types of materials, experimental systems and methods used in many studies. Here, indicate whether each material, system or method listed is relevant to your study. If you are not sure if a list item applies to your research, read the appropriate section before selecting a response.

Materials & experimental systems

n/a	Involved in the study
☒	☐ Antibodies
☒	☐ Eukaryotic cell lines
☒	☐ Palaeontology and archaeology
☒	☐ Animals and other organisms
☒	☐ Clinical data
☒	☐ Dual use research of concern
☒	☐ Plants

Methods

n/a	Involved in the study
☒	☐ ChIP-seq
☒	☐ Flow cytometry
☒	☐ MRI-based neuroimaging

Plants

Seed stocks	*Report on the source of all seed stocks or other plant material used. If applicable, state the seed stock centre and catalogue number. If plant specimens were collected from the field, describe the collection location, date and sampling procedures.*
Novel plant genotypes	*Describe the methods by which all novel plant genotypes were produced. This includes those generated by transgenic approaches, gene editing, chemical/radiation-based mutagenesis and hybridization. For transgenic lines, describe the transformation method, the number of independent lines analyzed and the generation upon which experiments were performed. For gene-edited lines, describe the editor used, the endogenous sequence targeted for editing, the targeting guide RNA sequence (if applicable) and how the editor was applied.*
Authentication	*Describe any authentication procedures for each seed stock used or novel genotype generated. Describe any experiments used to assess the effect of a mutation and, where applicable, how potential secondary effects (e.g. second site T-DNA insertions, mosiacism, off-target gene editing) were examined.*
